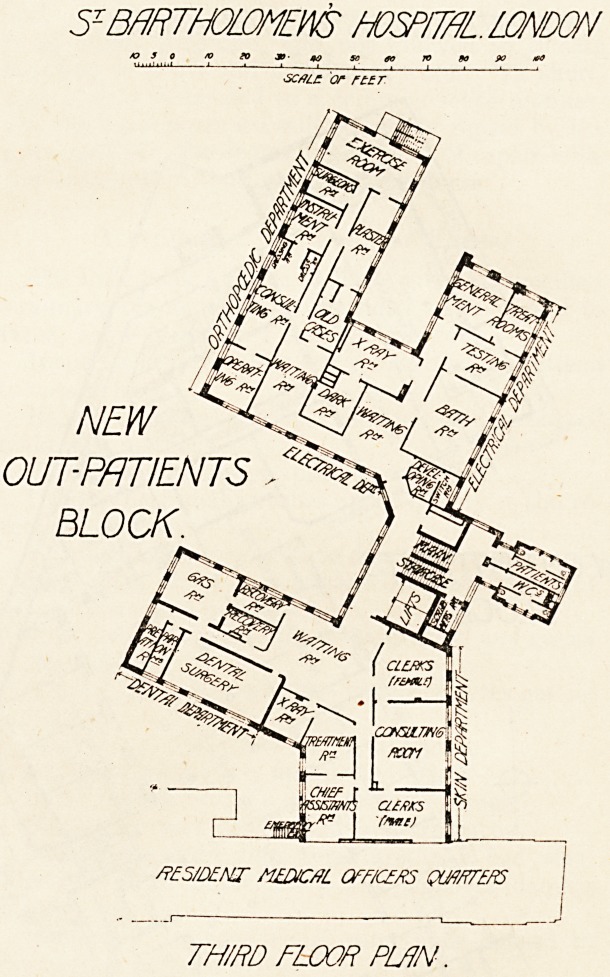# The New Out-Patient Department of St. Bartholomew's Hospital

**Published:** 1909-05-01

**Authors:** 


### HOSPITAL ADMINISTRATION.

### CONSTRUCTION AND ECONOMICS.

### THE NEW OUT-PATIENT DEPARTMENT OF ST. BARTHOLOMEW'S HOSPITAL.

DESCRIPTION OF THE BUILDINGS.

We publish this week plans of this important addition to the oldest hospital in London, which was formally opened by the Prince of Wales in July 1907 and is now in full occupation. The new buildings are entered from Giltspur Street and adjoin on the south the new Post Office extensions on the site of Christ's Hospital. On the north they adjoin other buildings of the hospital. A carriage entrance with footway at one side gives access for out-patients and casualties alike to an enclosed courtyard. Immediately on the left after passing the porter's lodge is the entrance for accidents. A large ante-room and two rooms of unequal size are devoted to the reception of accident and emergency cases. At the further end of the courtyard is a porch which gives access by two separate entrances, for men and women respectively, to a large waiting hall capable of seating upwards of 800 people. On all sides of this hall are the medical and surgical consulting rooms for casualties; besides these rooms there are two small operation rooms and two rooms for nurses.

GROUND FLOOR PLAN.

E. B. I'ANSON
ARCHITECT
7A LAURENCE POUNTNEY HILL E.C

FIRST FLOOR PLAN.

A passage at the back of the larger range of consulting rooms leads to the dispensary waiting hall, where patients are served with medicines, etc., at seven different windows, each bearing a notice stating the class of patients to be served, and there is a window reserved for urgent cases. In this hall is a small enclosed counter for the sale of bottles and other things which patients are expected to provide themselves. On leaving the dispensary patients reach the exit doorway by a separate passage, and thus all possibility of crossing other patients is avoided. The magnitude of the work carried out in this department may be gauged by the fact that during one day as many as 1,470 patients have been served. In proximity to this department is a set of a "cut-off" lobby are two closets for women and two closets and a urinal for men; also an orderly room for scrubbers. These offices are repeated on each upper floor. The surgery wards are eight in number, accommodating ten patients, and have attached a bathroom, kitchen, and nurses' room, with w.c. and sink room in a small tower with "cut-off" lobby.

The second floor is devoted to special departments. The department for diseases of women consists of a waiting room, consulting room, operation room, clinical room, dressing room, and laboratory. For the aural department there is a waiting room, surgeon's room, consulting room, operating room, recovery room, and clerks' room. The

SECOND FLOOR PLAN

THIRD FLOOR PLAN.

rooms for the out-patient and casualty sister, consisting of sitting room, kitchen, bedroom, bathroom, and w.c. There is also a small room for the night dresser and a w.c. and lavatory for nurses.

The staircase to the upper floors is in the centre of one side of the hall, and adjoining are two lifts for infirm or crippled patients.

The first floor is devoted to the medical and surgical out-patient work and to "surgery beds." The medical department comprises a large waiting room, consulting room, with small examining room attached, two rooms for clinical clerks, an assistant's room, and a dark room. On the surgical side is a waiting room, consulting room with two examination rooms, two rooms for dressers, an operation room with assistants' and surgeon's rooms attached. On the half landing of the staircase in a projecting tower with ophthalmic and throat departments are provided with a common waiting room. The ophthalmic department comprises a large consulting room, a small operation room, a room for septic cases, a spectacle room, and a dark room for ophthalmoscope work. For the throat department there is a consulting room, operation room, and recovery room, a room for clerks, and a dark room.

The third floor contains the departments for orthopædic work, electrical treatment, diseases of the skin, and dentistry.

The orthopædic department occupies the wing which on the floor below contains the department for diseases of women. It contains a waiting room, consulting room, operation room, instrument room, surgeon's room, room for physical exercise, plaster room, and a small room for seeing old cases.

The electrical department comprises a waiting room, two rooms for general treatment, testing room, bathroom, *x*-ray room with dark room attached, and a developing room with switch room attached.

The skin and dental departments are linked with a common waiting room. The skin department consists of a consulting room, with two clerks' rooms, one for male, the other for female patients, a room for the chief assistant, a room for treatment, and one for *x*-ray work. For the dental surgeon is provided a large surgery, a room for operations under gas, and two recovery rooms, and two small preparation rooms.

On the floor above is the clinical lecture theatre.

From a bare description of the accommodation it is difficult to realise the excellent way in which the problem set before the architect, Mr. E. B. I'Anson, has been dealt with. To contrive all these various departments, with their varying and, sometimes one would think, conflicting requirements, was no easy task; and it must be admitted that Mr. I'Anson has succeeded in producing a building that calls for little or no criticism. The lighting in particular of every room is excellent; whether the ventilation is equally efficient time alone will show. As regards the finishings, glazed tiles have been largely used on the walls, and the effect of the colour scheme is pleasant and at the same time appropriate to its purpose. There is no indication anywhere of extravagant outlay, while appearance has certainly not been neglected.

The large waiting hall on the ground floor is warmed and ventilated by a modification of the Plenum system, fans being provided for extraction; the remainder of the rooms are warmed by hot water ventilating radiators, and provided with extraction shafts fitted with electric fans, but ventilation by open windows is also available.

In addition to the extension described above there is a block containing rooms for the resident staff, facing Giltspur Street, and over the dispensary on the first floor a set of chemical laboratories, and on the floor above a complete new kitchen department.

## Figures and Tables

**Figure f1:**
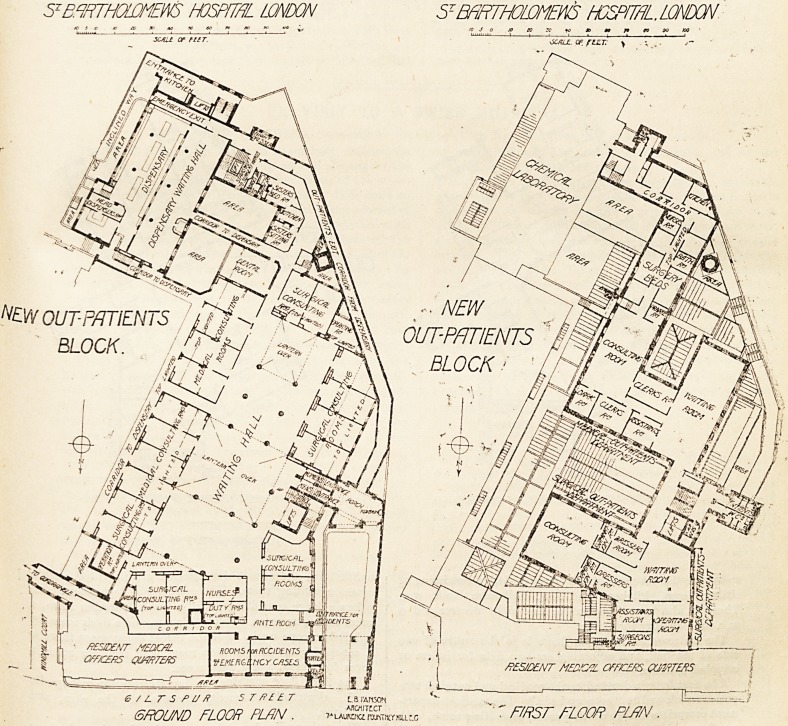


**Figure f2:**
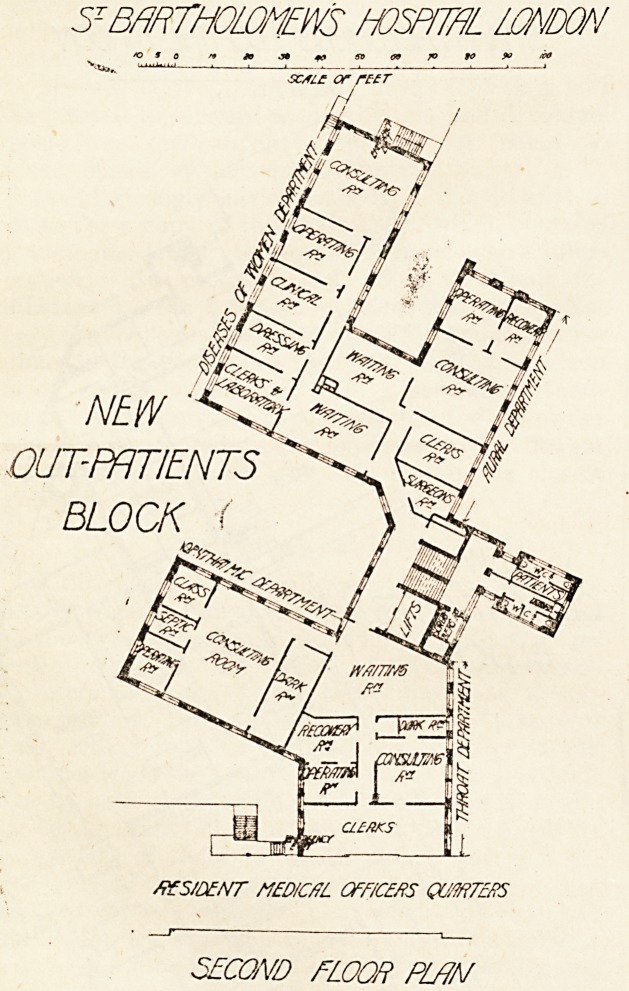


**Figure f3:**